# 2,5-Dimethyl-3-(4-methyl­phenyl­sulfin­yl)-1-benzofuran

**DOI:** 10.1107/S1600536812015450

**Published:** 2012-04-18

**Authors:** Hong Dae Choi, Pil Ja Seo, Uk Lee

**Affiliations:** a Department of Chemistry, Dongeui University, San 24 Kaya-dong Busanjin-gu, Busan 614-714, Republic of Korea; b Department of Chemistry, Pukyong National University, 599-1 Daeyeon 3-dong, Nam-gu, Busan 608-737, Republic of Korea

## Abstract

In the title compound, C_17_H_16_O_2_S, the 4-methyl­phenyl ring makes a dihedral angle of 88.28 (5)° with the mean plane [mean deviation = 0.009 (1) Å] of the benzofuran fragment. In the crystal, mol­ecules are linked by weak C—H⋯O and C—H⋯π inter­actions.

## Related literature

For background information and the crystal structures of related compounds, see: Choi *et al.* (2010*a* ▶,*b* ▶, 2012 ▶).
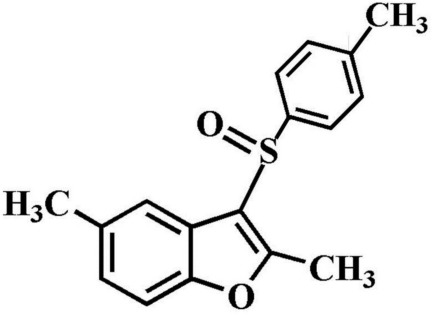


## Experimental

### 

#### Crystal data

C_17_H_16_O_2_S
*M* *_r_* = 284.36
Orthorhombic, 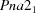

*a* = 13.072 (2) Å
*b* = 6.1790 (11) Å
*c* = 17.979 (3) Å
*V* = 1452.2 (4) Å^3^
*Z* = 4
Mo *K*α radiation
μ = 0.22 mm^−1^
*T* = 173 K
0.37 × 0.23 × 0.14 mm

#### Data collection

Bruker SMART APEXII CCD diffractometer
Absorption correction: multi-scan (*SADABS*; Bruker, 2009 ▶) *T*_min_ = 0.624, *T*_max_ = 0.746
13920 measured reflections
3587 independent reflections
3101 reflections with *I* > 2σ(*I*)
*R*_int_ = 0.036

#### Refinement

*R*[*F* ^2^ > 2σ(*F* ^2^)] = 0.036
*wR*(*F* ^2^) = 0.090
*S* = 1.03
3587 reflections
184 parameters
1 restraint
H-atom parameters constrained
Δρ_max_ = 0.20 e Å^−3^
Δρ_min_ = −0.24 e Å^−3^
Absolute structure: Flack (1983 ▶), 1729 Friedel pairs
Flack parameter: −0.01 (7)

### 

Data collection: *APEX2* (Bruker, 2009 ▶); cell refinement: *SAINT* (Bruker, 2009 ▶); data reduction: *SAINT*; program(s) used to solve structure: *SHELXS97* (Sheldrick, 2008 ▶); program(s) used to refine structure: *SHELXL97* (Sheldrick, 2008 ▶); molecular graphics: *ORTEP-3* (Farrugia, 1997 ▶) and *DIAMOND* (Brandenburg, 1998 ▶); software used to prepare material for publication: *SHELXL97*.

## Supplementary Material

Crystal structure: contains datablock(s) global, I. DOI: 10.1107/S1600536812015450/fy2054sup1.cif

Structure factors: contains datablock(s) I. DOI: 10.1107/S1600536812015450/fy2054Isup2.hkl

Supplementary material file. DOI: 10.1107/S1600536812015450/fy2054Isup3.cml

Additional supplementary materials:  crystallographic information; 3D view; checkCIF report

## Figures and Tables

**Table 1:** Hydrogen-bond geometry (Å, °) *Cg* is the centroid of the C2–C7 benzene ring.

*D*—H⋯*A*	*D*—H	H⋯*A*	*D*⋯*A*	*D*—H⋯*A*
C12—H12⋯O2^i^	0.95	2.60	3.334 (2)	134
C17—H17*B*⋯O1^ii^	0.98	2.52	3.387 (3)	148
C10—H10*B*⋯*Cg*^iii^	0.98	2.74	3.538 (3)	139

Symmetry codes: (i) 
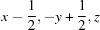
; (ii) 
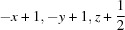
; (iii) 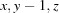.

**Fig. 1.**
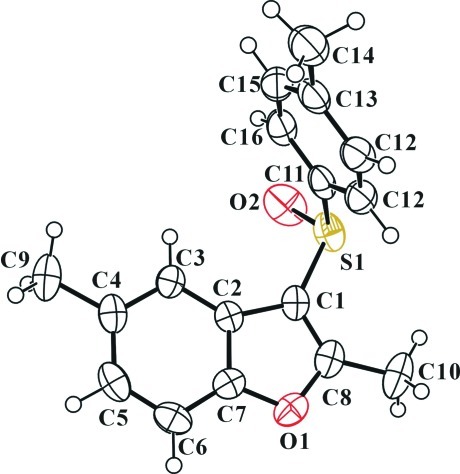
The molecular structure of the title compound with the atom numbering scheme. Displacement ellipsoids are drawn at the 50% probability level. H atoms are presented as small spheres of arbitrary radius.

**Fig. 2.**
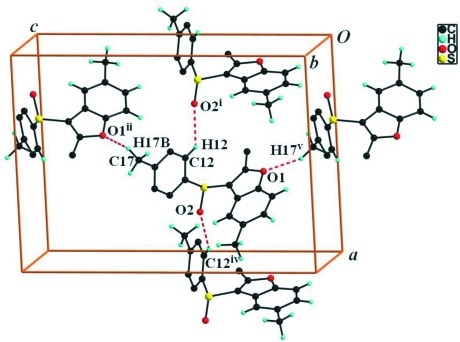
A view of the C—H···O hydrogen bonding interactions (dotted lines) in the crystal structure of the title compound. H atoms not participating in hydrogen bonding were omitted for clarity. [Symmetry codes: (i) x - 1/2, - y + 1/2, z ; (ii) - x + 1, - y + 1, z + 1/2; (iv) x + 1/2, - y + 1/2, z; (v) - x + 1, - y + 1, z - 1/2.]

**Fig. 3.**
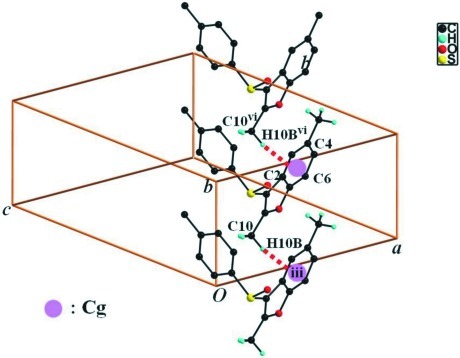
A view of the C—H···π interactions (dotted lines) in the crystal structure of the title compound. H atoms not participating in hydrogen bonding were omitted for clarity. [Symmetry codes: (iii) x, y - 1, z; (vi) x, y + 1, z.]

## supplementary crystallographic information

### Comment

As a part of our ongoing study of 2,5-dimethyl-1-benzofuran derivatives containing 3-(4-fluorophenylsulfinyl) (Choi *et al.*, 2010*a*), 3-(4-chlorophenylsulfinyl) (Choi *et al.*, 2010*b*) and 3-(4-bromophenylsulfinyl) (Choi *et al.*, 2012) substituents, we report herein the crystal structure of the title compound.

In the title molecule (Fig. 1), the benzofuran unit is essentially planar, with a mean deviation of 0.009 (1) Å from the least-squares plane defined by the nine constituent atoms. The dihedral angle between the 4–methylphenyl ring and the mean plane of the benzofurn fragment is 88.28 (5)°. In the crystal structure, molecules are connected by weak intermolecular C—H···O hydrogen bonds (Fig. 2 & Table 1) and C—H···π interactions (Fig. 3 & Table 1, Cg is the centroid of the C2–C7 benzene ring).

### Experimental

3-Chloroperoxybenzoic acid (77%, 291 mg, 1.3 mmol) was added in small portions to a stirred solution of 2,5-dimethyl-3-(4-methylphenylsulfanyl)-1-benzofuran (322 mg, 1.2 mmol) in dichloromethane (30 mL) at 273 K. After being stirred at room temperature for 4h, the mixture was washed with saturated sodium bicarbonate solution and the organic layer was separated, dried over magnesium sulfate, filtered and concentrated at reduced pressure. The residue was purified by column chromatography (hexane:ethyl acetate, 2:1 v/v) to afford the title compound as a colorless solid [yield 73%, m.p. 412–413 K; *R*_f_ = 0.44 (hexane:ethyl acetate, 2:1 v/v)]. Single crystals suitable for X-ray diffraction were prepared by slow evaporation of a solution of the title compound in benzene at room temperature.

### Refinement

All H atoms were positioned geometrically and refined using a riding model, with C—H = 0.95 Å for aryl and 0.98 Å for methyl H atoms. U_iso_(H) = 1.2*U*_eq_(C) for aryl and 1.5*U*_eq_(C) for methyl H atoms. The positions of methyl hydrogens were optimized rotationally.

### Crystal data

**Table:**

C_17_H_16_O_2_S	*F*(000) = 600
*M**_r_* = 284.36	*D*_x_ = 1.301 Mg m^−^^3^
Orthorhombic, *P**n**a*2_1_	Mo *K*α radiation, λ = 0.71073 Å
Hall symbol: P 2c -2n	Cell parameters from 4157 reflections
*a* = 13.072 (2) Å	θ = 2.3–24.8°
*b* = 6.1790 (11) Å	µ = 0.22 mm^−^^1^
*c* = 17.979 (3) Å	*T* = 173 K
*V* = 1452.2 (4) Å^3^	Block, colourless
*Z* = 4	0.37 × 0.23 × 0.14 mm

### Data collection

**Table:**

Bruker SMART APEXII CCD diffractometer	3587 independent reflections
Radiation source: rotating anode	3101 reflections with *I* > 2σ(*I*)
Graphite multilayer monochromator	*R*_int_ = 0.036
Detector resolution: 10.0 pixels mm^-1^	θ_max_ = 28.3°, θ_min_ = 2.3°
φ and ω scans	*h* = −17→16
Absorption correction: multi-scan (*SADABS*; Bruker, 2009)	*k* = −8→8
*T*_min_ = 0.624, *T*_max_ = 0.746	*l* = −23→23
13920 measured reflections	

### Refinement

**Table:**

Refinement on *F*^2^	Secondary atom site location: difference Fourier map
Least-squares matrix: full	Hydrogen site location: difference Fourier map
*R*[*F*^2^ > 2σ(*F*^2^)] = 0.036	H-atom parameters constrained
*wR*(*F*^2^) = 0.090	*w* = 1/[σ^2^(*F*_o_^2^) + (0.0445*P*)^2^ + 0.1605*P*] where *P* = (*F*_o_^2^ + 2*F*_c_^2^)/3
*S* = 1.03	(Δ/σ)_max_ = 0.001
3587 reflections	Δρ_max_ = 0.20 e Å^−^^3^
184 parameters	Δρ_min_ = −0.24 e Å^−^^3^
1 restraint	Absolute structure: Flack (1983), 1729 Friedel pairs
Primary atom site location: structure-invariant direct methods	Flack parameter: −0.01 (7)

### Special details

**Table:**

Geometry. All esds (except the esd in the dihedral angle between two l.s. planes) are estimated using the full covariance matrix. The cell esds are taken into account individually in the estimation of esds in distances, angles and torsion angles; correlations between esds in cell parameters are only used when they are defined by crystal symmetry. An approximate (isotropic) treatment of cell esds is used for estimating esds involving l.s. planes.
Refinement. Refinement of F^2^ against ALL reflections. The weighted R-factor wR and goodness of fit S are based on F^2^, conventional R-factors R are based on F, with F set to zero for negative F^2^. The threshold expression of F^2^ > 2sigma(F^2^) is used only for calculating R-factors(gt) etc. and is not relevant to the choice of reflections for refinement. R-factors based on F^2^ are statistically about twice as large as those based on F, and R- factors based on ALL data will be even larger.

### Fractional atomic coordinates and isotropic or equivalent isotropic displacement parameters (Å^2^)

**Table:**

	*x*	*y*	*z*	*U*_iso_*/*U*_eq_	
S1	0.68387 (4)	0.23443 (8)	0.43774 (3)	0.05093 (14)	
O1	0.60322 (10)	0.2839 (2)	0.22842 (8)	0.0447 (3)	
O2	0.79502 (12)	0.2480 (3)	0.45451 (10)	0.0708 (5)	
C1	0.66469 (12)	0.3081 (3)	0.34471 (11)	0.0379 (4)	
C2	0.70684 (12)	0.4873 (2)	0.30262 (10)	0.0332 (3)	
C3	0.77327 (12)	0.6586 (3)	0.31664 (11)	0.0372 (4)	
H3	0.8017	0.6784	0.3648	0.045*	
C4	0.79736 (14)	0.7997 (3)	0.25939 (12)	0.0424 (4)	
C5	0.75333 (16)	0.7692 (3)	0.18910 (12)	0.0482 (5)	
H5	0.7702	0.8677	0.1504	0.058*	
C6	0.68661 (15)	0.6026 (3)	0.17362 (10)	0.0464 (4)	
H6	0.6566	0.5850	0.1259	0.056*	
C7	0.66586 (12)	0.4626 (3)	0.23164 (10)	0.0373 (4)	
C8	0.60458 (13)	0.1927 (3)	0.29798 (12)	0.0419 (4)	
C9	0.87197 (17)	0.9835 (3)	0.27175 (15)	0.0580 (6)	
H9A	0.8709	1.0260	0.3243	0.087*	
H9B	0.8523	1.1073	0.2408	0.087*	
H9C	0.9410	0.9362	0.2582	0.087*	
C10	0.54077 (15)	−0.0042 (3)	0.30769 (17)	0.0602 (6)	
H10A	0.5488	−0.0592	0.3585	0.090*	
H10B	0.5626	−0.1152	0.2722	0.090*	
H10C	0.4688	0.0320	0.2988	0.090*	
C11	0.62497 (13)	0.4678 (3)	0.47906 (10)	0.0407 (4)	
C12	0.52077 (14)	0.4978 (3)	0.47194 (11)	0.0455 (4)	
H12	0.4809	0.3981	0.4440	0.055*	
C13	0.47520 (14)	0.6721 (4)	0.50530 (11)	0.0473 (4)	
H13	0.4035	0.6923	0.5000	0.057*	
C14	0.53160 (17)	0.8206 (3)	0.54687 (10)	0.0472 (5)	
C15	0.63554 (18)	0.7839 (4)	0.55478 (11)	0.0529 (5)	
H15	0.6753	0.8810	0.5838	0.063*	
C16	0.68289 (14)	0.6082 (4)	0.52116 (11)	0.0486 (5)	
H16	0.7543	0.5851	0.5271	0.058*	
C17	0.4802 (2)	1.0140 (4)	0.58236 (13)	0.0626 (6)	
H17A	0.5321	1.1069	0.6053	0.094*	
H17B	0.4320	0.9643	0.6205	0.094*	
H17C	0.4431	1.0960	0.5443	0.094*	

### Atomic displacement parameters (Å^2^)

**Table:**

	*U*^11^	*U*^22^	*U*^33^	*U*^12^	*U*^13^	*U*^23^
S1	0.0432 (2)	0.0524 (3)	0.0572 (3)	0.0094 (2)	0.0020 (2)	0.0228 (2)
O1	0.0372 (7)	0.0416 (7)	0.0554 (8)	0.0033 (5)	−0.0021 (6)	−0.0123 (6)
O2	0.0427 (8)	0.1013 (12)	0.0683 (12)	0.0268 (8)	−0.0071 (7)	0.0186 (9)
C1	0.0299 (8)	0.0329 (8)	0.0510 (10)	0.0048 (6)	0.0042 (7)	0.0049 (7)
C2	0.0281 (7)	0.0322 (7)	0.0393 (9)	0.0079 (6)	0.0039 (6)	0.0027 (7)
C3	0.0280 (8)	0.0372 (8)	0.0463 (10)	0.0022 (6)	0.0015 (7)	0.0002 (7)
C4	0.0330 (10)	0.0349 (9)	0.0592 (12)	0.0053 (7)	0.0113 (8)	0.0065 (8)
C5	0.0470 (12)	0.0485 (11)	0.0491 (12)	0.0105 (8)	0.0163 (9)	0.0134 (8)
C6	0.0486 (11)	0.0535 (11)	0.0372 (10)	0.0151 (9)	0.0034 (8)	0.0018 (8)
C7	0.0311 (8)	0.0378 (8)	0.0429 (9)	0.0078 (7)	0.0009 (7)	−0.0067 (7)
C8	0.0281 (9)	0.0335 (8)	0.0642 (12)	0.0062 (7)	0.0052 (8)	−0.0022 (8)
C9	0.0430 (11)	0.0409 (10)	0.0902 (17)	−0.0045 (9)	0.0144 (10)	0.0083 (10)
C10	0.0396 (10)	0.0380 (10)	0.1032 (19)	−0.0061 (8)	0.0087 (12)	−0.0085 (11)
C11	0.0326 (9)	0.0529 (10)	0.0367 (9)	−0.0013 (7)	0.0003 (7)	0.0173 (8)
C12	0.0329 (9)	0.0591 (11)	0.0445 (10)	−0.0029 (8)	−0.0024 (8)	0.0038 (9)
C13	0.0319 (9)	0.0677 (12)	0.0422 (10)	0.0019 (9)	0.0002 (8)	0.0051 (9)
C14	0.0505 (12)	0.0567 (11)	0.0344 (9)	−0.0048 (9)	0.0035 (8)	0.0120 (8)
C15	0.0536 (13)	0.0640 (13)	0.0411 (10)	−0.0184 (10)	−0.0084 (9)	0.0090 (9)
C16	0.0321 (9)	0.0685 (12)	0.0451 (11)	−0.0071 (9)	−0.0069 (8)	0.0181 (9)
C17	0.0751 (15)	0.0605 (13)	0.0521 (13)	−0.0027 (12)	0.0094 (11)	0.0035 (10)

### Geometric parameters (Å, º)

**Table:**

S1—O2	1.4864 (17)	C9—H9B	0.9800
S1—C1	1.751 (2)	C9—H9C	0.9800
S1—C11	1.795 (2)	C10—H10A	0.9800
O1—C8	1.372 (3)	C10—H10B	0.9800
O1—C7	1.376 (2)	C10—H10C	0.9800
C1—C8	1.354 (3)	C11—C16	1.378 (3)
C1—C2	1.450 (2)	C11—C12	1.381 (3)
C2—C3	1.392 (2)	C12—C13	1.369 (3)
C2—C7	1.392 (3)	C12—H12	0.9500
C3—C4	1.385 (3)	C13—C14	1.394 (3)
C3—H3	0.9500	C13—H13	0.9500
C4—C5	1.401 (3)	C14—C15	1.385 (3)
C4—C9	1.513 (3)	C14—C17	1.512 (3)
C5—C6	1.378 (3)	C15—C16	1.388 (3)
C5—H5	0.9500	C15—H15	0.9500
C6—C7	1.382 (3)	C16—H16	0.9500
C6—H6	0.9500	C17—H17A	0.9800
C8—C10	1.485 (2)	C17—H17B	0.9800
C9—H9A	0.9800	C17—H17C	0.9800
			
O2—S1—C1	108.60 (9)	H9A—C9—H9C	109.5
O2—S1—C11	106.86 (10)	H9B—C9—H9C	109.5
C1—S1—C11	97.17 (8)	C8—C10—H10A	109.5
C8—O1—C7	106.49 (14)	C8—C10—H10B	109.5
C8—C1—C2	107.41 (17)	H10A—C10—H10B	109.5
C8—C1—S1	122.59 (14)	C8—C10—H10C	109.5
C2—C1—S1	129.99 (14)	H10A—C10—H10C	109.5
C3—C2—C7	119.29 (16)	H10B—C10—H10C	109.5
C3—C2—C1	136.31 (17)	C16—C11—C12	120.55 (18)
C7—C2—C1	104.40 (15)	C16—C11—S1	119.83 (14)
C4—C3—C2	119.07 (17)	C12—C11—S1	119.51 (15)
C4—C3—H3	120.5	C13—C12—C11	119.63 (19)
C2—C3—H3	120.5	C13—C12—H12	120.2
C3—C4—C5	119.48 (17)	C11—C12—H12	120.2
C3—C4—C9	120.64 (19)	C12—C13—C14	121.49 (18)
C5—C4—C9	119.87 (18)	C12—C13—H13	119.3
C6—C5—C4	122.87 (18)	C14—C13—H13	119.3
C6—C5—H5	118.6	C15—C14—C13	117.8 (2)
C4—C5—H5	118.6	C15—C14—C17	121.5 (2)
C5—C6—C7	116.07 (18)	C13—C14—C17	120.7 (2)
C5—C6—H6	122.0	C14—C15—C16	121.39 (19)
C7—C6—H6	122.0	C14—C15—H15	119.3
O1—C7—C6	125.98 (17)	C16—C15—H15	119.3
O1—C7—C2	110.81 (16)	C11—C16—C15	119.12 (18)
C6—C7—C2	123.22 (17)	C11—C16—H16	120.4
C1—C8—O1	110.90 (15)	C15—C16—H16	120.4
C1—C8—C10	133.2 (2)	C14—C17—H17A	109.5
O1—C8—C10	115.87 (19)	C14—C17—H17B	109.5
C4—C9—H9A	109.5	H17A—C17—H17B	109.5
C4—C9—H9B	109.5	C14—C17—H17C	109.5
H9A—C9—H9B	109.5	H17A—C17—H17C	109.5
C4—C9—H9C	109.5	H17B—C17—H17C	109.5
			
O2—S1—C1—C8	133.66 (16)	C1—C2—C7—C6	179.67 (15)
C11—S1—C1—C8	−115.81 (15)	C2—C1—C8—O1	−0.62 (18)
O2—S1—C1—C2	−44.98 (18)	S1—C1—C8—O1	−179.53 (11)
C11—S1—C1—C2	65.56 (16)	C2—C1—C8—C10	−179.34 (18)
C8—C1—C2—C3	−178.81 (18)	S1—C1—C8—C10	1.8 (3)
S1—C1—C2—C3	0.0 (3)	C7—O1—C8—C1	0.57 (18)
C8—C1—C2—C7	0.41 (17)	C7—O1—C8—C10	179.54 (14)
S1—C1—C2—C7	179.21 (13)	O2—S1—C11—C16	−4.03 (17)
C7—C2—C3—C4	−0.3 (2)	C1—S1—C11—C16	−115.99 (15)
C1—C2—C3—C4	178.80 (17)	O2—S1—C11—C12	179.77 (14)
C2—C3—C4—C5	1.0 (2)	C1—S1—C11—C12	67.80 (15)
C2—C3—C4—C9	−177.99 (16)	C16—C11—C12—C13	1.8 (3)
C3—C4—C5—C6	−0.4 (3)	S1—C11—C12—C13	178.02 (15)
C9—C4—C5—C6	178.59 (17)	C11—C12—C13—C14	−0.3 (3)
C4—C5—C6—C7	−0.8 (3)	C12—C13—C14—C15	−1.3 (3)
C8—O1—C7—C6	179.98 (16)	C12—C13—C14—C17	179.07 (18)
C8—O1—C7—C2	−0.30 (17)	C13—C14—C15—C16	1.5 (3)
C5—C6—C7—O1	−178.81 (15)	C17—C14—C15—C16	−178.94 (19)
C5—C6—C7—C2	1.5 (3)	C12—C11—C16—C15	−1.7 (3)
C3—C2—C7—O1	179.32 (13)	S1—C11—C16—C15	−177.86 (14)
C1—C2—C7—O1	−0.07 (17)	C14—C15—C16—C11	0.0 (3)
C3—C2—C7—C6	−0.9 (2)		

### Hydrogen-bond geometry (Å, º)

Cg is the centroid of the C2–C7 benzene ring.

**Table:**

*D*—H···*A*	*D*—H	H···*A*	*D*···*A*	*D*—H···*A*
C12—H12···O2^i^	0.95	2.60	3.334 (2)	134
C17—H17*B*···O1^ii^	0.98	2.52	3.387 (3)	148
C10—H10*B*···*Cg*^iii^	0.98	2.74	3.538 (3)	139

Symmetry codes: (i) *x*−1/2, −*y*+1/2, *z*; (ii) −*x*+1, −*y*+1, *z*+1/2; (iii) *x*, *y*−1, *z*.
